# Region-based interaction detection in genome-wide case-control studies

**DOI:** 10.1186/s12920-019-0583-7

**Published:** 2019-12-30

**Authors:** Sen Zhang, Wei Jiang, Ronald CW Ma, Weichuan Yu

**Affiliations:** 1 Department of Chemical and Biological Engineering, The Hong Kong University of Science and Technology,, Kowloon, Hong Kong, China; 2 Department of Electronic and Computer Engineering, The Hong Kong University of Science and Technology, Kowloon, Hong Kong, China; 3 Department of Medicine and Therapeutics, The Chinese University of Hong Kong, Shatin, Hong Kong, China

**Keywords:** GWAS, Statistical interaction detection, Region-based method, LD contrast test

## Abstract

**Background:**

In genome-wide association study (GWAS), conventional interaction detection methods such as BOOST are mostly based on SNP-SNP interactions. Although single nucleotides are the building blocks of human genome, single nucleotide polymorphisms (SNPs) are not necessarily the smallest functional unit for complex phenotypes. Region-based strategies have been proved to be successful in studies aiming at marginal effects.

**Methods:**

We propose a novel region-region interaction detection method named RRIntCC (region-region interaction detection for case-control studies). RRIntCC uses the correlations between individual SNP-SNP interactions based on linkage disequilibrium (LD) contrast test.

**Results:**

Simulation experiments showed that our method can achieve a higher power than conventional SNP-based methods with similar type-I-error rates. When applied to two real datasets, RRIntCC was able to find several significant regions, while BOOST failed to identify any significant results. The source code and the sample data of RRIntCC are available at http://bioinformatics.ust.hk/RRIntCC.html.

**Conclusion:**

In this paper, a new region-based interaction detection method with better performance than SNP-based interaction detection methods has been proposed.

## Background

Genome-wide association study (GWAS) has served as an important tool to investigate the relationship between genomic variants and human traits [1]. The genetic variants investigated in GWAS are mainly single nucleotide polymorphisms (SNPs). SNPs are single nucleotide variants whose genotypes are not fixed in the population and exhibit diversities among different individuals. Most GWAS analysis protocols follow the single-locus test procedures aimed at detecting the marginal effects of SNPs [2, 3]. However, it’s well recognized that genetic variants work synergistically through certain pathogenic pathways [4]. The interactions among SNPs are not guaranteed to be discovered by marginal effect detection, especially for SNPs with weak marginal effects but strong interaction effects [5]. Many methods have been developed to address this problem [4, 6], including PLINK [7], BOOST [5], MDR [8], ReliefF [9], BEAM [10], and LD contrast test [11].

An important problem in SNP-SNP interaction detection is the stringent threshold when considering multiple testing correction. For marginal effect detection, a SNP can only be considered as significant when its corresponding *p*-value is at the order of 10^−8^ (assuming we use Bonferroni correction). In SNP-SNP interaction detection, the threshold has to go down further to the order of 10^−14^. As a result, interactions with weak or moderate effect sizes might remain undiscovered.

In this paper, we proposed a region-based interaction detection method to address this problem. Region-based methods have been successful in marginal effect detection [12, 13]. The basic idea is to group the effects of nearby SNPs together and test their aggregation rather than investigating the elements separately. The benefit is two-folds: Firstly, the size and the number of regions are controllable. We can achieve the balance between the resolution of the results and the statistical significance threshold after Bonferroni correction. Secondly, the effect size might be enhanced by taking the whole region into account. SNPs are the basic genomic units. Neverthless, they are not necessarily the functional units of diseases. Different SNP mutations in a gene can all lead to changes of protein functions. Therefore, grouping different SNPs together provides a possible alternative.

To group different SNP-SNP pairs together, the key is to quantitatively measure and account for the relationships between different SNP-SNP pairs. To the best of our knowledge, no existing method is available to test region-region interactions for case-control studies, where we only have two groups of people: healthy people (controls) and people with the investigated disease (cases). Although Ma et al. [14] proposed a region-based interaction detection method to analyze continuous traits based on the linear regression model, it is not easy to extend their method to the case-control setting due to the difficulty of deriving the covariances of test statistics under the logistic regression model that is commonly used in case-control studies. In this paper, we use the LD contrast test method instead of the logistic regression in interaction detection. We derive the correlation coeffcients of the correpsonding SNP-SNP interaction test statistics. Then we further extend region-based methods to the case-control setting by accounting for the covariances between SNP-based test statistics. We name this method RRIntCC (region-region interaction detection for case-control studies). Experiment results illustrate that RRIntCC achieves a higher power than conventional SNP-SNP interaction detection methods at the same type-I-error rate.

## Methods

Here we propose a novel region-based interaction detection method for genome-wide case-control studies that utilizes SNP-based interaction test statistics and their covariances. LD contrast test is adopted to measure SNP-based interaction effects. We derive the covariance of LD contrast test statistics, which enables a robust aggregation of SNP-SNP interactions within a region pair. The determination of regions comes from gene definitions or BOOST results.

### Genomic data formats

There are two alleles for almost every base pair (bp) position in the human genome, one from the maternal chromosome and the other from the paternal chromosome. A combination of the two alleles is denoted as a genotype of this bp position. SNPs are defined as the base pairs that could exhibit different genotype values in different individuals. Normally a SNP only has two possible allele values in the population, one major allele with a higher probability (denoted as B), and one minor allele (denoted as b). Correspondingly, there exist three genoytpes for a typical SNP, i.e., BB, Bb and bb, where Bb is called a heterogeneous genotype and the rest two are called homogeneous genotypes. GWAS uses microarrays to generate SNP genotype data. In SNP data analysis, we use 0/1/2, 0/1/1, and 0/0/1 for BB/Bb/bb as the encoding scheme for additive, dominant, and recessive genetic models, respectively. A more flexible strategy is to estimate the effects of three genotypes independently, at the price of an increased degree of freedom. Allele data could also be used for analysis, with 0/1 as the numerical values of major/minor alleles. However, statistical inference needs to be performed in advance to retrieve allele information from original genotype data, which is called haplotype phasing in the GWAS community. In this paper, we focus on the analysis of genotype data.

### LD contrast test for SNP interaction detection

Current interaction detection methods are mainly based on the deviation from additive effect by assuming a linear or logistic regression model. Nevertheless, this approach is not necessarily the most powerful method due to the uncertainty of underpinning biochemical mechanisms. Linkage disequilibrium (LD) contrast test provides another valuable perspective to investigate this problem. Empirical studies have shown that LD contrast test can achieve higher power than logistic regression under certain disease models for case-control studies [6]. In this paper, LD contrast test is adopted to generate SNP-based interaction test statistics because of its clear statistical meaning and mathematical simplicity.

LD represents the statistical association between two genetic loci with allele values, defined as the deviation from the independence of two SNPs (*A* and *B*)

1 $$ LD = p(A,B)-p(A)p(B).  $$

To avoid the ambiguity caused by haplotype phasing, composite LD (CLD) which only requires genotype data is commonly used to approximate LD. CLD is defined as [15]:

2 $$\begin{array}{@{}rcl@{}} &&\ \ \ CLD = p_{AB}+p'_{AB}-2p(A)p(B) \\ with&&\ \left\{ \begin{array}{ll} p_{AB} = P^{AB}_{AB}+\frac{1}{2}\left(P^{AB}_{Ab}+P^{AB}_{aB}+P^{AB}_{ab}\right) \\ p'_{AB} = P^{AB}_{AB}+\frac{1}{2}\left(P^{AB}_{Ab}+P^{AB}_{aB}+P^{aB}_{Ab}\right) \end{array} \right., \end{array} $$

where the subscript and the superscript represent two gametes that are passed to offsprings and *P* denotes the probability of the specific gamete combination. CLD could be regarded as a simplified version of phasing to facilitate the analysis based on genotype data. The statistical properties of CLD have been well studied [16, 17]. One important fact is that CLD corresponds to the sample correlation coefficient $\hat {r}$ of genotype values under the additive model,

3 $$ {{} \begin{aligned} \hat{r}_{genotype} \,=\, \frac{CLD}{\sqrt{p(1-p) + D_{A}}\sqrt{q(1-q) + D_{B}}} \approx \frac{CLD}{\sqrt{p(1-p)}\sqrt{q(1-q)}}, \end{aligned}}  $$

where *p*=*p*(*A*),*q*=*p*(*B*),*D*_*A*_ and *D*_*B*_ represent Hardy-Weinberg disequilibriums, i.e. *D*_*A*_=*p*_*AA*_−*p*^2^(*A*), *D*_*B*_=*p*_*BB*_−*p*^2^(*B*). *D*_*A*_ and *D*_*B*_ are nearly 0 in GWAS datasets after quality control.

A similar result holds for the original LD and allele values,

4 $$ \hat{r}_{allele} = \frac{LD}{\sqrt{p(1-p)}\sqrt{q(1-q)}}.  $$

Therefore, CLD could also be viewed as an approximation of LD by using the correlation coefficient of 0/1/2 genotype data under the addtive model to replace that of 0/1 allele values, at the price of implicitly conducting phasing with equal probabilities for two-allele combinations.

Suppose two SNPs work synergistically to contribute to the same pathways, they are less likely to be separated during recombination and will be inherited together to offsprings in the case group. As a result, the SNP-SNP pattern should be different between patients and healthy people. Therefore, checking the difference of LD patterns between cases and controls provides an alternative way to detect interaction. LD contrast test was proposed to statistically test this difference [11]. The test statistic based on CLD has the following form:

5 $$ \chi^{2} = \frac{\left(\hat{CLD}_{AB}^{case}-\hat{CLD}_{AB}^{control}\right)^{2}} {Var\left(\hat{CLD}_{AB}^{case}\right)+Var\left(\hat{CLD}_{AB}^{control}\right)},  $$

which follows a 1-df *χ*^2^ distribution under the null hypothesis that there is no LD difference between cases and controls.

### Covariance between SNP interactions

The key issue in the aggregation of individual SNP-SNP interaction effects is the correction of inflated effect sizes caused by the correlations among individual test statistics. The fact that LD is actually the sample covariance of two SNPs is leveraged to derive the correlation coefficients of LD contrast test statistics.

Suppose two SNP pairs (*X,Y*) and (*U,V*) have interactions with contrast LDs

6 $$ \left\{ \begin{array}{ll} \Delta LD_{XY} = \hat{cov}(X, Y | case) - \hat{cov}(X, Y | control) \\ \Delta LD_{UV} = \hat{cov}(U, V | case) - \hat{cov}(U, V | control) \end{array}\right..  $$

The corresponding LD contrast test statistics read:

7 $$ T_{XY}=\frac{\Delta \hat{LD_{XY}}}{\sqrt{Var(\Delta\hat{LD_{XY}})}}\ \text{and}\ \ T_{UV}=\frac{\Delta \hat{LD_{UV}}}{\sqrt{Var(\Delta\hat{LD_{UV}})}}.  $$

The covariance of the two test statistics reads:

8 $$ {{} \begin{aligned} cov(T_{XY}, T_{UV}) \approx \frac{cov(\Delta\hat{LD_{XY}}, \Delta\hat{LD_{UV}})}{\sqrt{cov(\Delta\hat{LD_{XY}}, \Delta\hat{LD_{XY}})cov(\Delta\hat{LD_{UV}}, \Delta\hat{LD_{UV}})}}. \end{aligned}}  $$

In GWAS, it’s commonly assumed that population samples are independent. Under this assumption, we can derive the following theorems.

#### Theorem 1.

The covariance of contrast LDs can be decomposed into components from cases and controls separately,

9 $$ {{} \begin{aligned} cov(\Delta LD_{XY}, \Delta LD_{UV}) = & cov[\hat{cov}(X,Y|case),\hat{cov}(U,V|case)] \\ &+ cov[\hat{cov}(X,Y|control),\hat{cov}(U,V|control)]. \end{aligned}}  $$

#### Proof 1.

*ΔLD* is the difference of the two sample covariances in cases and controls. By the linear property of covariance, *cov*(*ΔLD*_*XY*_,*ΔLD*_*UV*_) can be decomposed into four covariances of two sample covariances. Because individuals are assumed to be independent, the two terms with one sample covariance from cases and the other from controls are 0. Therefore, Theorem 1 holds. □

#### Theorem 2.

The covariance of sample covariances reads

10 $$ cov\left[\hat{cov}(X, Y), \hat{cov}(U,V)\right]=\frac{1}{n}\left(\delta_{4}-\delta_{2}+\frac{\sigma_{2}+\tau_{2}}{n-1}\right),  $$

where

$$\begin{array}{@{}rcl@{}} \delta_{4} &=& E\left[(X-EX)(Y-EY)(U-EU)(V-EV)\right], \\ \delta_{2} &=& cov(X,Y)cov(U,V), \\ \sigma_{2} &=& cov(X,U)cov(Y,V), \\ \tau_{2} &=& cov(X,V)cov(Y,U). \end{array} $$

#### Proof 2.

The covariance of sample covariances can be rewritten as

11 $$ {\begin{aligned} &cov\left[\hat{cov}(X, Y), \hat{cov}(U,V)\right]\\ &\quad= cov\left[{\textstyle \frac{1}{2n(n-1)}}\sum_{i=1}^{n}\sum_{j=1}^{n}(X_{i}-X_{j})(Y_{i}-Y_{j}), {\textstyle \frac{1}{2n(n-1)}}\right.\\& \quad\left.\sum_{i=1}^{n}\sum_{j=1}^{n}(U_{i}-U_{j})(V_{i}-V_{j})\right] \\ &\quad= {\textstyle \frac{1}{4n^{2}(n-1)^{2}}}\sum_{j=1}^{n}\sum_{j=1}^{n}\sum_{k=1}^{n}\sum_{l=1}^{n}\\ &\quad cov\left[(X_{i}-X_{j})(Y_{i}-Y_{j}),\ (U_{k}-U_{l})(V_{k}-V_{l})\right]. \end{aligned}}  $$

We consider the following four conditions. (1) *i*=*j* or *k*=*l*. (2) *i*≠*j,i*≠*k,i*≠*l,j*≠*k,j*≠*l* and *k*≠*l*. (3) *i*≠*j* and { *i*=*k,j*=*l* or *i*=*l*,*j*=*k*}. (4) *i*≠*j,k*≠*l*, and { *i*=*k* or *i*=*l* or *j*=*k* or *j*=*l*}. The basic covariance unit in (11) can be rewriten as

12 $$ {{} \begin{aligned} &cov\left\{\left[(X_{i}-EX)-(X_{j}-EX)\right]\left[(Y_{i}-EY)-(Y_{j}-EY)\right]\right., \\ &\left. \left[(U_{k}\,-\,EU)\,-\,(U_{l}\,-\,EU)\right]\left[(V_{k}-EV)-(V_{l}-EV)\right]\right\}. \end{aligned}}  $$

There are 2*n*^3^−*n*^2^,*n*(*n*−1)(*n*−2)(*n*−3),2*n*(*n*−1) and 4*n*(*n*−1)(*n*−2) items for the four conditions respectively. We can further separate (12) into 16 components and calculate their values under different conditions. The derivation is straightforward. Our conclusion thus holds. □

#### Theorem 3.

The sample mean of (*X*−*EX*)(*Y*−*EY*)(*U*−*EU*)(*V*−*EV*)is an asympototically unbiased estimator of *δ*_4_,

13 $$ {{} \begin{aligned} &E\left[\frac{1}{n}\sum_{i=1}^{n}\left(X_{i}-\frac{1}{n}\sum_{j=1}^{n}X_{j}\right) \left(Y_{i}-\frac{1}{n}\sum_{j=1}^{n}Y_{j}\right)\right.\\ &\left. \left(U_{i}-\frac{1}{n}\sum_{j=1}^{n}U_{j}\right) \left(V_{i}-\frac{1}{n}\sum_{j=1}^{n}V_{j}\right)\right] \\ &= \left(1\,-\,\frac{4}{n}\,+\,\frac{6}{n^{2}}\,-\,\frac{3}{n^{3}}\right)\delta_{4} \,+\, \left[\frac{2(n-1)}{n^{2}}\,-\,\frac{3(n-1)}{n^{3}}\right]\left(\delta_{2}+\sigma_{2}\,+\,\tau_{2}\right) \xrightarrow{n\rightarrow\infty} \delta_{4}. \end{aligned}}  $$

#### Proof 3.

Equation (13) can be rewritten as

14 $$ {\begin{aligned} &\sum_{i=1}^{n}E\left\{\left[(X_{i}-EX)-\left(\frac{1}{n}\sum_{j=1}^{n}X_{j}-EX\right)\right]\right. \\ &\quad \left[(Y_{i}-EY)-(\frac{1}{n}\sum_{j=1}^{n}Y_{j}-EY)\right] \\ &\quad \left[(U_{i}-EU)-\left(\frac{1}{n}\sum_{j=1}^{n}U_{j}-EU\right)\right]\\&\quad \left. \left[(V_{i}-EV)-\left(\frac{1}{n}\sum_{j=1}^{n}V_{j}-EV\right)\right]\right\}. \end{aligned}}  $$

Again (14) can be separated into 16 components which are solvable under the independence assumption. The rest of the proof is omitted due to page limit. □

By integrating (8-13), the covariance of the LD contrast test statistics can be estimated. Note that the variance of the standardized LD contrast test statistic is approximately 1,

15 $$ {} Var(T_{XY})\,=\,Var\left[\frac{\Delta \hat{LD_{XY}}}{\sqrt{Var(\Delta\hat{LD_{XY}})}}\right] \approx \frac{Var(\Delta\hat{LD_{XY}})}{Var(\Delta\hat{LD_{XY}})}=1.  $$

Therefore, the covariance of *T*_*XY*_ and *T*_*UV*_ can be reduced to the corresponding correlation coefficients,

16 $$ corr(T_{XY},T_{UV}) \approx cov(T_{XY}, T_{UV}).  $$

### The test statistic for region-based interactions

To aggregate SNP-SNP interaction test statistics, a minimum *p*-value based method is adopted. In detail, we assume a multivariate normal distribution *MVN*(0,*Σ*) for the observed test statistics *z*_*i*_,*i*=1,2,...,*k*_1_*k*_2_, where *k*_1_ and *k*_2_ are the number of SNPs in the two regions. The covariance matrix *Σ* is estimated using (8-13).

Then the region-based *p*-value is defined as the probability that we observe a value that is larger than the largest absolute value of SNP-SNP interaction test statistics under *MVN*(0,*Σ*). Denote the absolute value of the test statistic related to the minimum *p*-value as *T*:

17 $$ T=\left|\Phi^{-1}\left(\frac{min(p_{i},\ i=1,2,..,k_{1}k_{2}}{2}\right)\right|.  $$

Then the *p*-value for this region-region interaction reads,

18 $$ {\begin{aligned} p_{\text{region-region}} &= Pr\left[max(|z_{i}|,\ i=1,2,..,k_{1}k_{2})\right.\\ & \left.\geq T\ |\ z_{i}\sim MVN(0,\Sigma)\right] \\ &= 1 - Pr\left[max(|z_{i}|,\ i=1,2,..,k_{1}k_{2})\right.\\ & \left.< T\ |\ z_{i}\sim MVN(0,\Sigma)\right] \\ &= 1 - Pr\left[\{|z_{i}|<T,\ i=1,2,..,k_{1}k_{2}\} |\ z_{i}\right.\\ &\left.\sim MVN(0,\Sigma)\right]. \end{aligned}}  $$

In this paper, We use the results of GBOOST [18], the GPU version of BOOST, to specify candidate regions. The regions could also be selected by checking potential pathogenic pathways or protein-protein interaction networks.

## Results

We conducted simulations under various settings to examine whether the proposed method can correctly control type-I-error rates and outperform SNP-based methods in terms of statistical power. To mimic real LD patterns, we picked all genotyped SNPs from two genomic regions (A and B) with intensive LD patterns in the dataset from Myocardial Infarction Genetics Consortium (MIGen) [19]. Region A is of size 157.874 kbp, located in chromosome 1, with 34 genotyped SNPs inside and 9 tag SNPs selected by haploview. Region B is of size 267.528 kbp, located in chromosome 3, with 50 genotyped SNPs and 10 tag SNPs.

We developed the software RRIntCC in C++. The source code of RRIntCC is available at http://bioinformatics.ust.hk/RRIntCC.html. The results of RRIntCC and SNP-based methods were compared for empirical power experiments. We further applied RRIntCC to MIGen and a renal complication dataset of type 2 diabetes (T2D) patients. RRIntCC reported several significant region pairs in both datasets while conventional SNP-based interaction detection tools failed to identify any SNP pairs.

### Type-I-Error rate control

**Fig. 1:**
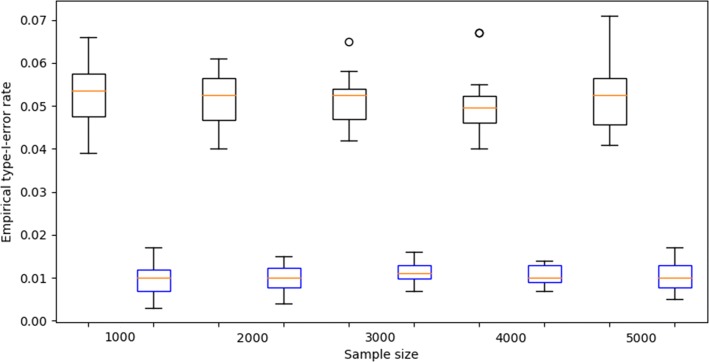
The boxplots of empirical type-I-error rates at the significant levels of 0.05 (black) and 0.01 (blue)

For type-I-error rate evaluation, we randomly selected 1000, 2000, 3000, 4000, and 5000 samples from MIGen dataset and maintained their genotype values to preserve the LD patterns. Phenotype values for the randomly picked samples were assigned using a Bernoulli distribution with equal probabilities for case and control disease status. 1000 simulations were run for each sample size to determine the empirical type-I-error rates under two commonly used significance levels, i.e. 0.05 and 0.01. We repeat the experiment 20 times to examine the robustness of empirical type-I-error rates. As shown in Fig 1, simulations of empirical type-I-error rates indicated that the results of RRIntCC are not inflated at given significance levels.

### Empirical statistical power

For power evaluation, phenotype values were generated using the public software GWASimulator [20], which uses haplotype information to simulate LD structure and produces phenotype values according to preset disease prevalence, causal SNPs and interactions with certain effect sizes. In total, 12084 haplotypes of these two regions were generated by PLINK [7]. We performed 1000 simulations for 1000, 2000, 3000, 4000, and 5000 samples, respectively. Results of original LD contrast test (LDCont) and GBOOST were also given for comparison.

GWASimulator simulated genotypes of all SNPs in the two regions, while only the tag SNPs were analyzed. Even though non-tag SNPs could be selected as causal SNPs, we can still observe interaction effects between tag SNPs due to LD between tag SNPs and non-tag SNPs. We designed six experimental settings with different tag status and allele frequencies for the causal interacted SNP pair. The effect sizes were determined by the relative risk ratio. The increment of relative risk ratio by observing one disease allele was set as $\sqrt {2}$, so that the ratios for genotype combinations 1/1, 1/2, 2/1, and 2/2 were 2, $2\sqrt {2}, 2\sqrt {2}$, and 4, respectively. The results are summarized in Table 1. Under all settings, RRIntCC achieves a higher power than LDCont. GBOOST outperforms RRIntCC and LDCont when the MAFs of both causal SNPs are large. However, when the MAF of even one causal SNP goes down, the power of GBOOST drops dramatically and RRIntCC is the most powerful method under such settings. Even in the cases where both MAFs are large, RRIntCC is still valuable when sample size is small. The results support the use of our region-based interaction detection method in GWAS studies, especially considering that GWAS datasets usually have quite limited sample sizes compared to the huge number of SNPs.

**Table 1:** Empirical statistical power results

		1000	2000	3000	4000	5000
19(0.424) ∼28(0.414)	RRIntCC	**0.247**	0.564	0.806	0.913	0.975
	LDCont	0.205	0.524	0.778	0.888	0.968
	GBOOST	0.240	**0.624**	**0.872**	**0.961**	**0.994**
19(0.424) ∼22*(0.413)	RRIntCC	**0.255**	0.545	0.814	0.924	0.979
	LDCont	0.214	0.489	0.793	0.905	0.969
	GBOOST	0.218	**0.609**	**0.885**	**0.968**	**0.998**
15(0.067) ∼22*(0.413)	RRIntCC	**0.244**	**0.548**	**0.772**	**0.880**	**0.964**
	LDCont	0.188	0.496	0.724	0.849	0.953
	GBOOST	0.058	0.211	0.411	0.559	0.731
23*(0.067) ∼22*(0.413)	RRIntCC	**0.307**	**0.631**	**0.882**	**0.954**	**0.986**
	LDCont	0.264	0.574	0.856	0.942	0.975
	GBOOST	0.088	0.272	0.548	0.713	0.838
15(0.067) ∼25(0.094)	RRIntCC	**0.116**	**0.266**	**0.398**	**0.551**	**0.667**
	LDCont	0.072	0.204	0.323	0.480	0.612
	GBOOST	0.012	0.060	0.108	0.224	0.285
23*(0.067) ∼25(0.094)	RRIntCC	**0.110**	**0.282**	**0.502**	**0.638**	**0.790**
	LDCont	0.081	0.220	0.428	0.576	0.729
	GBOOST	0.025	0.064	0.161	0.259	0.397

The indices are the order of SNPs in their corresponding regions, * means this SNP is a tag SNP, and the values in the brackets denote minor allele frequencies (MAFs).

### Experiment using real datasets

We applied our method to the dataset of Myocardial Infarction Genetics Consortium (MIGen) with 649370 genotyped SNPs and 2967/3075 cases/controls, and the renal complication dataset collected in Hong Kong with 1257031 SNPs and 882/2231 cases/controls. Current computation capability cannot support whole-genome interaction analysis using LD contrast test. Instead, GBOOST [18] was first used as probes to generate region-pairs qfor region-based interaction analysis. We adopted 5×10^−10^ as a suggestive *p*-value threshold to screen out SNP pairs that are unlikely to be associated. The remaining SNP pairs were clumped into regions with size 200 kbp, which is roughly the size of typical genes. After identifying the ranges of clumped regions, all genotyped SNPs in MIGen dataset were mapped into these regions. For computation efficiency, the maxmium number of SNPs in each region was set to be 31, so that the total number of SNP-SNP interactions within each region pair was controlled below 1000. The choice of this number is arbitrary. In case that the real number of SNPs inside a region is larger than this limit, we randomly choose 31 SNPs to represent this region.

Table 2 lists the top four SNP pairs found by GBOOST in the MIGen dataset and their corrected family-wise error rates (cFWER). None of them can pass the Bonferroni-corrected *p*-value threshold. Moreover, even the smallest *p*-value is 100 times larger than the threshold. Table 3 lists the top four region pairs found by RRIntCC. One region pair, chr3: [177577480, 177777480] ∼ chr7: [81695481, 81895481], passes the Bonferroni-corrected *p*-value threshold. The second and third region pairs share the same region in chr3 and overlap in the region in chr20, which indicates that these two region pairs actually refer to only one region pair with size larger than the preset 200 kbp. Therefore, we further analyze the region interaction between chr3: [187498383, 187698383] with size 200 kbp and chr20: [39109460, 39444799] with size 335.339 kbp, leading to a cFWER of 0.0536. Multiple genes, including CACNA2D1, DGKG, AK057298, TOP1, BC035080, PLCG1, ZHX3, LPIN3, and EMILIN3, are located in these two region pairs. CACNA2D1 has been found to be involved in cardiomyopathy pathway [21, 22]. Besides, ZHX3 is reported to be associated with left ventricle wall thickness [23]. Both ZH3 and EMILIN3 are reported to be associated with resting heart rate [24]. The regions identified by RRIntCC might provide clues for factors affecting myocardial infarction risks.

**Table 2:** Top four SNP pairs found by GBOOST in the MIGen dataset

SNP pairs	*p*-value	cFWER
rs4678428 (chr3) ∼ rs9961565 (chr18)	2.588×10^−11^	>1
rs17626606 (chr5) ∼ rs11190346 (chr10)	2.679×10^−11^	>1
rs11925209 (chr3) ∼ rs1501909 (chr5)	3.006×10^−11^	>1
rs6930292 (chr6) ∼ rs114313 (chr6)	3.026×10^−11^	>1

**Table 3:** Top four region pairs found by RRIntCC in the MIGen dataset

region pairs	*p*-value	cFWER
chr3: [177577480, 177777480] ∼ chr7: [81695481, 81895481]	1.652×10^−10^	0.0186
chr3: [187498383, 187698383] ∼ chr20: [39244799, 39444799]	5.363×10^−10^	0.0603
chr3: [187498383, 187698383] ∼ chr20: [39109460, 39309460]	7.497×10^−10^	0.0843
chr2: [184236258, 184436258] ∼ chr13: [29010198, 29210198]	7.835×10^−9^	0.8814

We also applied GBOOST and RRIntCC to the renal complication dataset. GBOOST has no significant finding, while RRIntCC found one region pair, chr12: [103040398, 103240398] and chr15: [33102602, 33302602], with a cFWER of 0.00382. Two genes, PAH and FMN1, are involved in this region pair. Both PAH and FMN1 were reported to be related to kidney disorders [25][26], which implies a potentially target pathway for the study of renal complications in patients with T2D.

## Discussion

There still remain several issues that could be improved in our method. First, the computation complexity of calculating the covariance matrix is *O*(*n*^2^), which is unacceptable for whole genome analysis. Second, the genomic resolution has been sacrificed by replacing SNPs with regions. One potential remedy is to extend statistical fine mapping methods for interaction detection to determine the leading SNP pairs within the significant region pairs.

## Conclusions

In this paper, we proposed a region-based interaction detection method named RRIntCC. We derived the correlation coefficients between SNP-SNP interaction test statistics by using LD contrast test. We aggregated SNP-SNP interaction test statistics by assuming a multi-variate normal distribution with the estimated covariance matrix to account for the potential intensive LD pattern within the regions. By using region-based strategy, we reduced the total number of tests and were therefore able to use a less stringent Bonferroni-corrected *p*-value threshold. Simulation results support that our region-based strategy outperforms SNP-based method in terms of statistical power at similar type-I-error rates.

## Data Availability

The data of Myocardial Infarction Genetics Consortium are publicly available from The database of Genotypes and Phenotypes (dbGaP), accession number: phs000294.v1.p1. The data of renal complication in T2D patients are are available from the Theme-based Research Scheme (T12-402/13N) of the Hong Kong Research Grant Council (RGC) but restrictions apply to the availability of these data, which were used under license for the current study, and so are not publicly available. Data are however available from the authors upon reasonable request and with permission of the Theme-based Research Scheme (T12-402/13N).

## Abbreviations

bp: Base Pair
cFWER: Corrected family-wise error rate
CLD: Composite linkage disequilibrium
GWAS: Genome-wide association study
LD: Linkage disequilibrium
MAF: Minor allele frequency
MIGen: Myocardial infarction genetics consortium
RRIntCC: Region-region interaction detection for case-control studies
SNP: Single nucleotide polymorphism
T2D: Type II diabetes
